# Closed-Loop Neuroprosthesis for Reach-to-Grasp Assistance: Combining Adaptive Multi-channel Neuromuscular Stimulation with a Multi-joint Arm Exoskeleton

**DOI:** 10.3389/fnins.2016.00284

**Published:** 2016-06-23

**Authors:** Florian Grimm, Alireza Gharabaghi

**Affiliations:** Division of Functional and Restorative Neurosurgery, Department of Neurosurgery, and Centre for Integrative Neuroscience, Eberhard Karls University TuebingenTuebingen, Germany

**Keywords:** functional electrical stimulation, robot-assisted rehabilitation, feedback, virtual reality, individualized therapy, hemiparesis, upper-limb assistance, hybrid assistance

## Abstract

Stroke patients with severe motor deficits cannot execute task-oriented rehabilitation exercises with their affected upper extremity. Advanced rehabilitation technology may support them in performing such reach-to-grasp movements. The challenge is, however, to provide *assistance as needed*, while maintaining the participants' commitment during the exercises. In this feasibility study, we introduced a closed-loop neuroprosthesis for reach-to-grasp assistance which combines adaptive multi-channel neuromuscular stimulation with a multi-joint arm exoskeleton. Eighteen severely affected chronic stroke patients were assisted by a gravity-compensating, seven-degree-of-freedom exoskeleton which was attached to the paretic arm for performing reach-to-grasp exercises resembling activities of daily living in a virtual environment. During the exercises, adaptive electrical stimulation was applied to seven different muscles of the upper extremity in a performance-dependent way to enhance the task-oriented movement trajectory. The stimulation intensity was individualized for each targeted muscle and remained subthreshold, i.e., induced no overt support. Closed-loop neuromuscular stimulation could be well integrated into the exoskeleton-based training, and increased the task-related range of motion (*p* = 0.0004) and movement velocity (*p* = 0.015), while preserving accuracy. The highest relative stimulation intensity was required to facilitate the grasping function. The facilitated range of motion correlated with the upper extremity Fugl-Meyer Assessment score of the patients (*p* = 0.028). Combining adaptive multi-channel neuromuscular stimulation with antigravity assistance amplifies the residual motor capabilities of severely affected stroke patients during rehabilitation exercises and may thus provide a customized training environment for patient-tailored support while preserving the participants' engagement.

## Introduction

The majority of stroke survivors remain dependent on others for activities of daily living due to a dysfunctional upper extremity (Jørgensen et al., 1999; Dobkin, 2005; Feigin et al., 2008). However, when clinically meaningful improvements are achieved, they correlate positively with the dose of therapy (Lohse et al., 2014; Pollock et al., 2014). Many studies, therefore, aimed to further increase the number of task-oriented exercises by applying assistive robotic technologies for stroke rehabilitation; often resulting in improved arm/hand function and muscle strength, albeit respective trials have, as yet, provided only low-quality evidence (Mehrholz et al., 2015). However, critical voices attribute technology-assisted improvements such as these to unspecific influences like increased enthusiasm for novel interventions on the part of both patients and therapists (Kwakkel and Meskers, 2014). In the same vein, when compared to dose-matched conventional physiotherapy, robot-assisted training showed no clinically relevant, *additional* benefits in controlled trials (Lo et al., 2010; Klamroth-Marganska et al., 2014).

This dilemma might be illustrated, for example, by the most advanced commercially available training system for the upper limb; an *active* robotic exoskeleton with seven actuated axes (i.e., degrees of freedom) that provides antigravity support for the paretic arm and enables patients with severe impairment to perform task-oriented movements (Klamroth-Marganska et al., 2014; Kwakkel and Meskers, 2014; Brauchle et al., 2015). This device provided slightly more functional gain for the participating stroke survivors, but was less effective in restoring arm strength than conventional therapy (Klamroth-Marganska et al., 2014), probably because it was too supportive when providing *assistance as needed* during the exercises (Chase, 2014; Brauchle et al., 2015).

In this context, neurophysiological parameters might constitute a means of preserving patient engagement and of avoiding under-challenge. Other studies applied surface electromyography to infer the person's intention to perform a particular movement and used it as a control signal for robotic assistance (Maciejasz et al., 2014). For severely impaired stroke patients, however, who might benefit most from robotic therapy (Klamroth-Marganska et al., 2014; Brauchle et al., 2015), this physiological parameter might be inadequate due to paralysis and/or abnormally co-activated muscles (Wright et al., 2014). Novel robotic devices, therefore, move only when the brain is most responsive to the feedback by the multi-joint exoskeleton (Brauchle et al., 2015). More specifically, patients control the rehabilitation robots with their brain signals—i.e., via motor imagery-related oscillations of the ipsilesional cortex—within the framework of a brain-robot interface (BRI) for stroke rehabilitation (Brauchle et al., 2015). Although, this technique makes it possible to successfully link three-dimensional robotic training to the participants' own effort, some findings also suggest that sustained brain self-regulation for brain-controlled robotic training is challenging and that it may even be characterized by a significant association with the experience of frustration for the participants (Fels et al., 2015). This potential drawback of connecting rehabilitation exercises to physiological parameters might possibly be overcome, if the resources available for coping with the mental load, that occurs in conjunction with BRI technology, are taken into consideration and when the task difficulty is adjusted accordingly (Naros and Gharabaghi, 2015; Bauer and Gharabaghi, 2015a,b; Naros et al., 2016a). At the same time, however, a direct comparison of the perceived workload of BRI tasks and classical rehabilitation exercises on the basis of voluntary muscle contraction suggested that the experience of frustration and over-challenge was task-independent, thus supporting the notion that the perceived workload was influenced by the characteristics of the individual subject (Fels et al., 2015).

Accordingly, *assistance as needed* has to be individually adjusted during stroke rehabilitation and, if not used precisely, is constantly confronted with the dangers of both under- and over-challenge, no matter what assistive technology is applied. However, current assisted approaches usually take an all-or-nothing approach, e.g., by providing active robotic guidance to complete a movement as soon as the patient failed to reach the defined goal (Klamroth-Marganska et al., 2014); or by triggering functional electrical stimulation (FES) for overt muscle contraction as soon as a predefined physiological state (recorded with EMG or EEG) is achieved (Howlett et al., 2015).

More *targeted* assistance might, therefore, be necessary during these rehabilitation exercises to maintain engagement without compromising the patients' motivation; i.e., providing support as little as possible and as much as necessary. Along these lines, we explored an alternative approach to classical assistive technology in this feasibility study. Instead of applying standard robot-guided rehabilitation or triggered FES, we minimized the robotic assistance to pure antigravity support while providing performance-dependent, neuromuscular electrical stimulation with subthreshold modulation of individual upper limb muscles. Notably, the robotic assistance was passive, and the electrical stimulation was non-functional, i.e., elicited no overt movement. We hypothesized, however, that this combined, closed-loop approach leads to a wider range of motion than any one of these assistive tools by itself.

## Methods

Eighteen stroke patients (female/male: 6/12; right/left hemispheric stroke: 13/5, ischemic/hemorrhagic: 13/5; mean age: 56 ± 9.8 [34 69] years) in the chronic phase after stroke (78 ± 55.3 [8 244] months) presented with a severe and persistent hemiparesis. The modified upper extremity Fugl-Meyer-Assessment score (i.e., mean motor UE-FMA score without coordination, speed, and reflexes; Naros and Gharabaghi, 2015) of our group of patients was 15.6 ± 4.9 [9 25]. This study, which was approved by the ethical review committee of the local medical faculty, involved two sessions of reach-to-grasp training with a multi-joint exoskeleton attached to the paretic arm. Each session lasted approximately 30 min. and consisted of 150 trials. The exoskeleton, virtual reality, and task design have been described in detail elsewhere (Grimm et al., 2016) and are cited here.

### Exoskeleton and virtual reality

We used a commercially available (Armeo Spring, Hocoma, Volketswil, Switzerland) rehabilitation exoskeleton for shoulder, elbow and wrist joints with seven axes (i.e., degrees of freedom), providing antigravity support for the paretic arm and registration of movement kinematics and grip force. The un-weighing was realized via two springs incorporated into the device. This device could be used to make individual adjustments e.g., of gravity compensation, thereby supporting patients with severe impairment in performing task-oriented practice within a motivating virtual environment. We extended these features in-house by using the real-time sensor data of the exoskeleton to display a three-dimensional multi-joint visualization of the user's arm in virtual reality. This entailed capturing the angles of all arm joints and the grip force from a shared memory block using a file mapping communication protocol. The virtual arm engine was programmed in a Microsoft XNA™ framework. The arm model utilized by the engine was constructed as a meshed bone-skin combination with 54 bones (3Ds Max 2010™, Autodesk). The measured joint angles (accuracy 0.1°) and grip forces of the device were used to modify the bone-vectors of the meshed model according to the movements of the user, thereby providing online closed-loop feedback. The joint angles of the exoskeleton were directly represented in virtual reality, whereas the grip forces were augmented to feedback natural hand function. Prior to each session, participants were instructed to perform a natural reach-to-grasp movement during the task by using distal (elbow) rather than proximal (shoulder) movements. The three-dimensional visualization of the arm was then applied during each task as an implicit online feedback of the movement since explicit information can disrupt motor learning in stroke patients (Boyd and Winstein, 2004; Cirstea and Levin, 2007). Various virtual training paradigms were designed to allow for different rehabilitation exercises resembling activities of daily living.

### Task design

In this study, participants performed self-paced, three-dimensional (in x-, y-, and z-direction) reach-to-grasp movements in virtual space. Patients could interact within the virtual space via the virtual arm representation described above. The position of the virtual arm changed in real-time according to the patient's arm position tracked by the orthosis. The grasping and releasing of the virtual ball was performed by applying force to the grip sensor and opening the hand, respectively. The relationship between the force applied to the grip sensor and the virtual movement was adjusted individually to each user.

After system setup, the exercise was presented on a screen in front of the patient. This exercise consisted of a transfer movement, i.e., a ball had to be grasped in virtual space and transferred to a basket. The position of the ball and the basket in space and in relation to each other was randomly distributed in x- (left to right/ right to left), y- (up to down/down to up), or z-direction (front to back/ back to front). After presenting the objects in virtual space, the patients had to move the virtual hand toward the presented ball. The movements were self-paced and no distinct timing was given. After grasping the ball, three-dimensional transfer movements toward a basket were necessary, i.e., the ball had to be grasped, carried to a distant basket and then released again. The timing for this transfer movement was self-induced. The virtual hand could interact with the ball as soon as it entered a defined range around the latter. The ball changed its color according to the hand position (white: out of range, green: possible to grasp, yellow: possible to transfer, red: possible to release). After releasing the ball in the basket, the next exercise started by presenting the next ball randomly distributed in virtual space.

### Closed-loop neuromuscular stimulation

**Figure 1:**
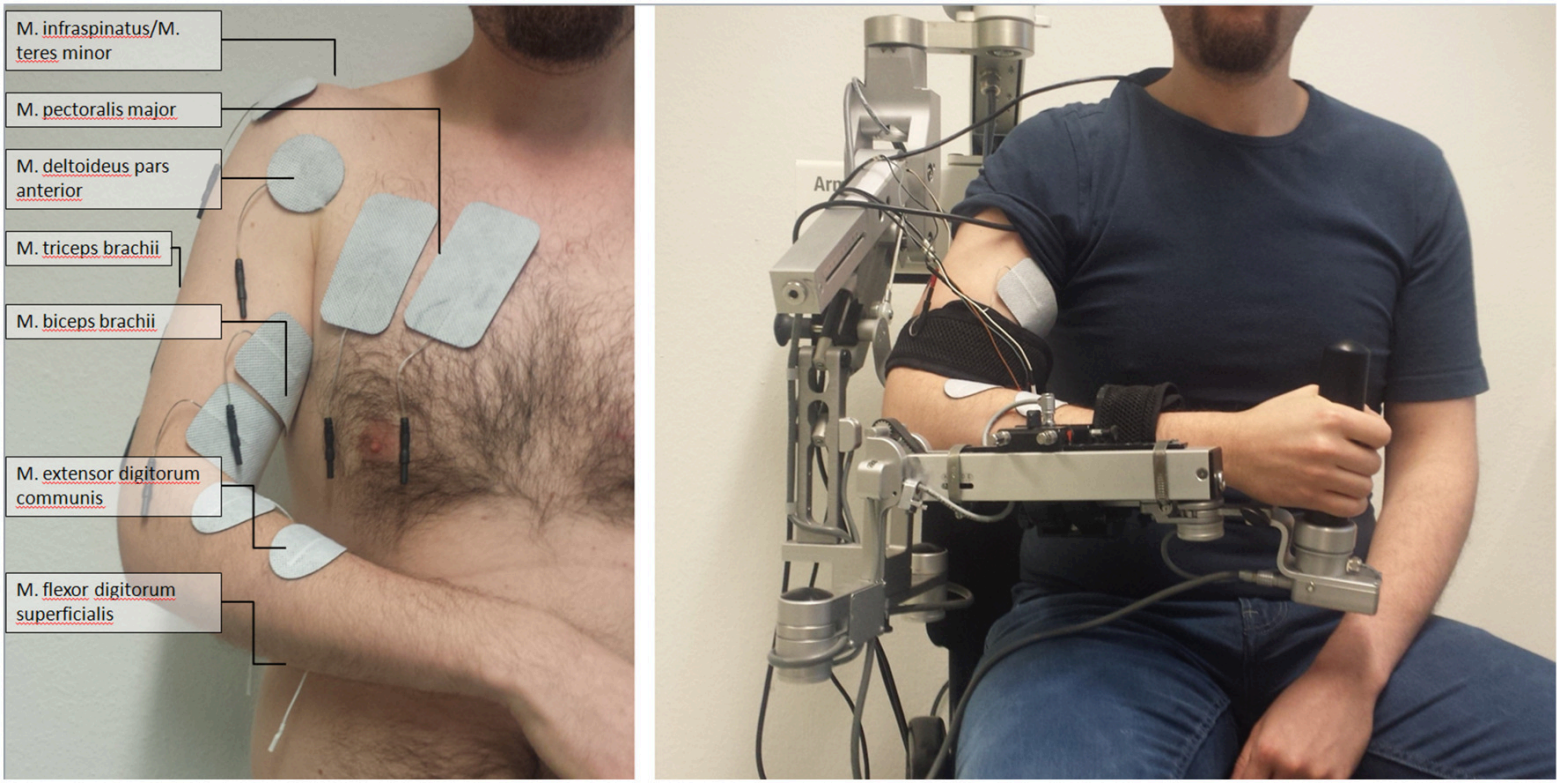
**Set-up of multi-channel neuromuscular stimulation integrated into a gravity-compensating, seven-degree-of-freedom exoskeleton**.

We integrated a neuromuscular electrical stimulation device in the exoskeleton-based training environment (Rehastim, 8-channel stimulator, Hasomed GmbH, Magdeburg, Germany), and applied biphasic square impulses (frequency: 30 Hz, pulse width: 500 μs). The stimulation intensity of this integrated neuroprosthesis was updated in a closed-loop, real-time iteration at 60 Hz via a controller area network (CAN)/universal serial bus (USB) port using a custom-made algorithm. This made it possible to stimulate seven different muscles / muscle groups relevant for reaching and grasping, while the output current was adapted continuously for each of them: M. extensor digitorum communis, M. flexor digitorum superficialis, M. biceps brachii, M. triceps brachii, M. pectoralis major, M. infraspinatus/M. teres minor (i.e., muscle group), M. deltoideus pars anterior. In pairs of antagonist muscles/muscle groups, only one of them was stimulated at the same time; i.e., either M. extensor digitorum communis or M. flexor digitorum superficialis, either M. biceps brachii or M. triceps brachii, either M. pectoralis major or M. infraspinatus/M. teres minor. This resulted—together with the M. deltoideus pars anterior—in up to four simultaneously stimulated, co-activated muscles/muscle groups (Figure 1).

**Figure 2:**
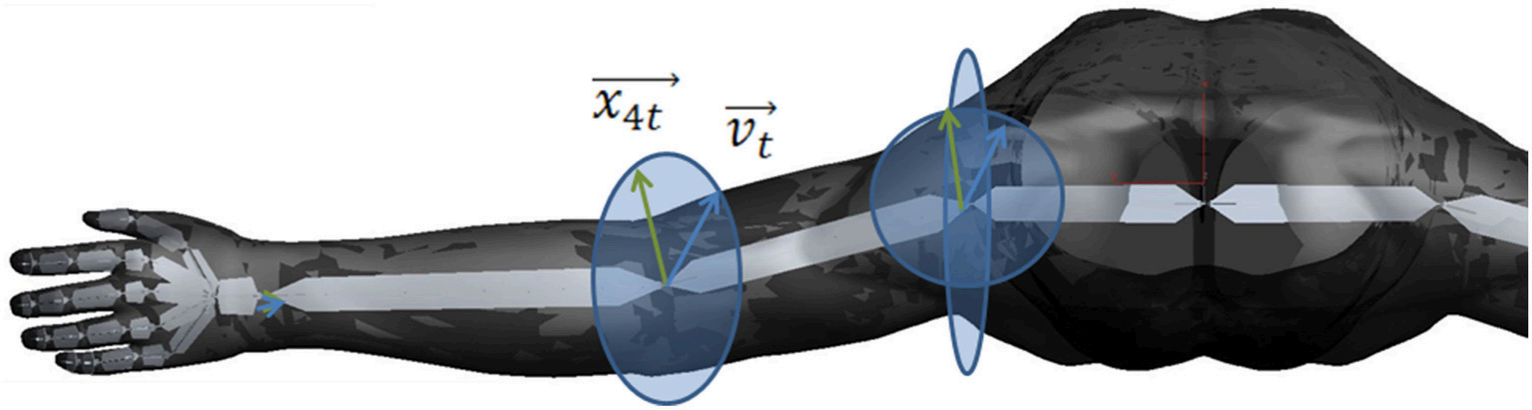
**Biomechanical movement model on the basis of the vector positions of the virtual arm**. The vector v_t_ (blue) is representing the target vector necessary to reach the object. The vector x_4t_ (green) is representing the movement vector of stimulating the M. biceps brachii in the elbow joint. The ellipses represent the movement radius.

**Figure 3:**
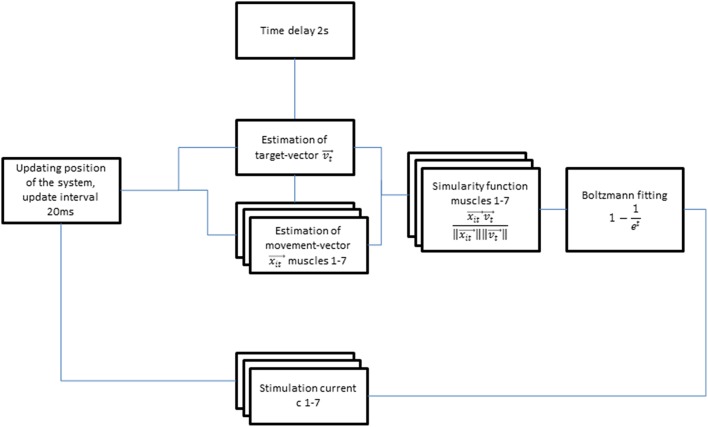
**Flow diagram of the autoadaptive stimulation algorithm**.

According to a biomechanical movement model (Figure 2) on the basis of the vector positions of the virtual arm, the neuromuscular stimulation pattern and intensity was calculated (Figure 3).

More specifically, the target vector and the estimated movement vector of each individual muscle group were calculated on the basis of the real-time arm position measured by the exoskeleton, while using the cosine similarity functions between the two vectors for weighting. This function gives a maximum output of 1 when the target vector and the estimated movement vector of the corresponding muscle group are pointing in the same direction. When the vectors point in opposite directions, the function would result in a negative output and is then set to zero, i.e., resulting in no stimulation. For positive outputs, i.e., when target vector and estimated movement vector point in the same direction, the stimulation amplitudes are calculated by multiplying the weighting of the muscle vectors with a Boltzmann-fitting of the time course of a ramping stimulation toward maximum stimulation strength (Equation 1). This stimulation began with a 2 s delay to avoid instability of the induced movement.

(1) $$\begin{array}{cc} \begin{array}{l} c_{i}(t)=\left(1-\frac{1}{e^{t}}\right)c_{i\;max}\frac{\vec{x_{\iota t}}\vec{v_{t}}}{\left\|\vec{x_{\iota t}}\right\|\left\|\vec{v_{t}}\right\|}\\ c_{i}\in R_{0+}\;:\text{ stimulation current, muscle group i}\\ x_{\iota t}:\text{ estimated movement vector, muscle group i}\\ \vec{v_{t}}\;:\text{ estimated target vector} \end{array} & \;(1) \end{array}$$

Each patient performed two consecutive exoskeleton-supported training sessions—one with and one without concurrent stimulation—in randomized order. Both the exoskeleton and the maximum stimulation intensity (Stim_max_) were individually calibrated: The exoskeleton was adjusted to provide optimized gravity compensation for every joint and to allow for goal-directed movements in three-dimensional space. The gravity compensation was provided by a spring mechanism of the orthotic device, which was calibrated individually to balance the weight of the patient's paretic arm. Thereby, the exoskeleton was adjusted to the corresponding functional anatomy of the participant before each session. Particularly, the shoulder position, forearm, and upper arm length were considered for the adjustments.

For calibration of the stimulation intensity, the different muscles/muscle groups where identified anatomically before applying the self-sticking FES electrodes (Han-Sen GmbH, Hamburg, Germany; 50 mm diameter and 50 × 80 mm). The electrode positions were optimized by subsequent test stimulation. Since all participants suffered from severe upper limb impairment, prolonged supra-motor threshold stimulation was perceived as painful and ineffective and was therefore not applied. The Stim_max_ for each muscle group was empirically determined as the output current perceived as comfortable and approaching the motor threshold, but remaining still subthreshold. The motor threshold was identified by a visible joint movement. Each muscle group was stimulated separately before the training session to determine the individual maximum stimulation strength.

### Outcome measures

The kinematic assessment included movement smoothness, temporal efficiency, and range of motion (volume). Movement smoothness was captured by calculating changes of movement direction along an optimal direct path toward the targets, by estimating the distance function between the hand-position and the final endpoint, and by calculating the second derivative of the function to determine the number of turning points for each task (Cirstea et al., 2006). In order to avoid compensatory shoulder movements the stimulation patterns for shoulder and biceps activation were equally distributed for an inbound trajectory. Temporal efficiency was captured as the time required to complete each task, and as the mean and peak velocity of the hand between the targets while calculating their distance for x-, y-, and z-directions in virtual units (vu). The overall range of motion (volume, vu^3^) was measured as the complete space covered during the exercises. The range of sensor data from the grip-sensor was estimated as the mean change in grip pressure. The range of shoulder, upper arm, and forearm movement was measured in degrees.

The maximum (Stim_max_) and mean stimulation amplitude (in mA) was calculated per channel, i.e., muscle group. In addition, we captured the stimulation period for different stimulation intensities, i.e., <25% Stim_max_, 25–50% Stim_max_, >50–75% Stim_max_, >75% Stim_max._

### Statistics

Statistical analysis was performed on a Matlab 2010b Engine. For paired data points a *t*-test for paired samples was performed. The significance level was set at *p* = 0.0125 for all tests after conservative Bonferroni correction. Correlation coefficients r and respective *p*-values were calculated between the overall range of motion and the UA-FMA score.

## Results

The three-dimensional reach-to-grasp exercises of this study could be completed only with the help of assistive technology. None of the patients was able to complete grasping exercises in unsupported conditions, i.e., they all scored 0 out of a possible 4 points in the related FMA sub-scores (“grasp cylinder,” “grasp tennis ball”). However, neuromuscular stimulation alone was not sufficient in our severely impaired patient group, i.e., none of the targeted muscles was stimulated in a functionally relevant way to allow for overt muscle contraction. The patients were, therefore, unable to perform a reach-to-grasp movement *per se*, even when neuromuscular stimulation was applied. Instead, the multi-joint antigravity assistance was essential to facilitate the goal-oriented grasping exercises in the 3D-virtual environment and required commitment from the patients.

Fifteen of the patients were able to complete all 150 trials in each session. The amount of training had to be reduced for three patients, two of whom completed 75 trials and the third 50 trials in each session. This resulted in a group mean of 135 trials (±32.2808, [50 150]). The reach-to-grasp direction was randomly distributed (x: 44.4 ± 12.1 vu, y: 45.1 ± 11.2 vu, z: 44.1 ± 12.6 vu).

**Figure 4:**
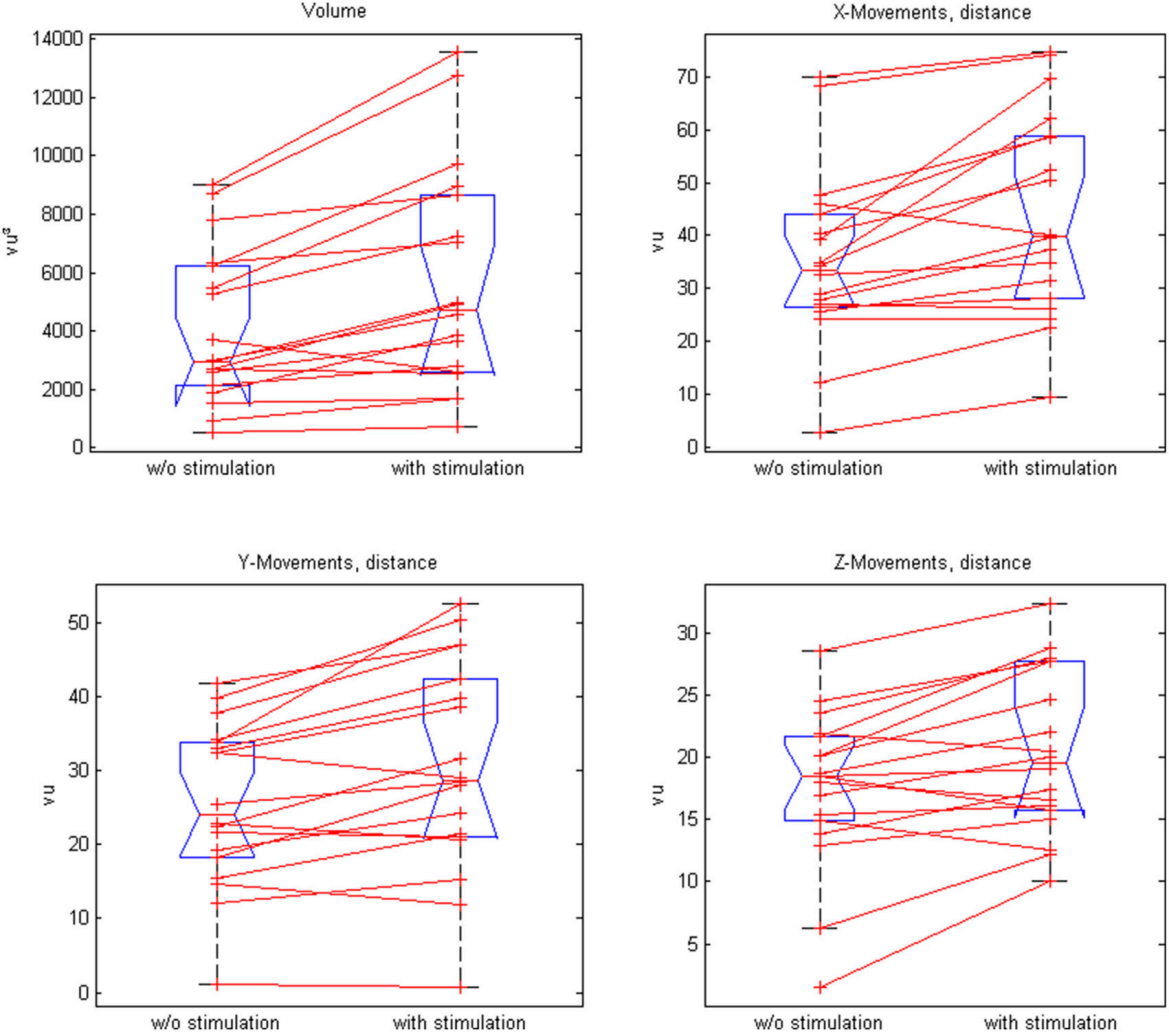
**Comparison of paired plots for mean values of the range of motion (volume) and distances in x-, y-, and z- direction with and without stimulation**.

Closed-loop neuromuscular stimulation could be integrated well into the exoskeleton-based training; this neuroprosthesis increased the task-related range of motion (ROM) in 16 out of 18 participants as well as the mean ROM of all patients (*p* = 0.0004). More distant targets in the virtual training space were achieved in all three x-, y-, z-directions and the participants were able to perform longer transfer movements, i.e., inter-target distances (Table 1, Figure 4).

**Table 1:** **Virtual training space with and without stimulation**.

	**Volume (vu^3^)**	**Distance between targets (vu)**	**x-Movements distance (vu)**	**y-Movements distance (vu)**	**z-Movements distance (vu)**
Training space in virtual units (with and w/o stim)	4877 [548 13539]	27.8 ± 10.9 [1.650.4]	39.6 ± 18.0 [2.9 74.6]	28.0 ± 12.7 [0.8 52.6]	18.9 ± 6.45 [1.6 32.4]
Neuroprosthesis (with stimulation)	5667 [7366 13538.8]	30.7 ± 12.2 [7.2 50.4]	44.1 ± 19.2 [9.5 74.6]	30.5 ± 14.3 [0.8 52.6]	20.3 ± 6.5 [10.1 32.4]
Orthosis (w/o stimulation)	4087 [5488 9023]	24.86 ± 9.58 [1.6 39.8]	35.1 ± 16.7 [2.9 69.8]	25.4 ± 10.9 [1.1 41.9]	17.5 ± 6.4 [1.6 28.6]
Significance level, *p*-value(^*^significant)	0.0004^*^	0.0001^*^	0.0007^*^	0.001^*^	0.002^*^

**Figure 5:**
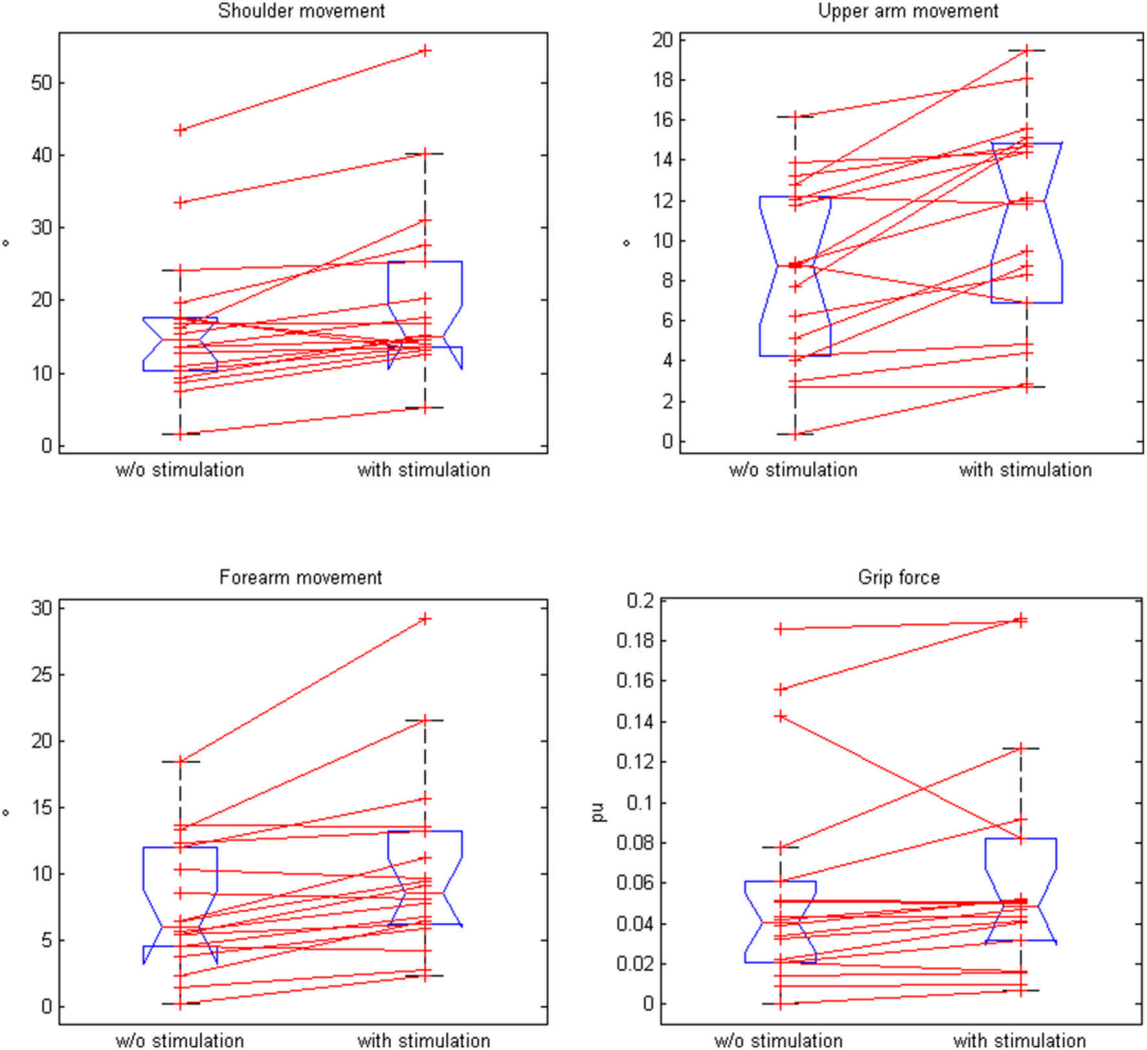
**Comparison of paired plots for mean values of kinematic data (for shoulder movement, upper arm movement, forearm movement, and grip force) with and without stimulation**.

Moreover, the movement range of the shoulder, upper arm, and forearm increased significantly in the neuroprosthesis condition, while the grip pressure showed a trend (Table 2, Figure 5). The range of motion correlated with the upper extremity Fugl-Meyer Assessment score of the patients for both the antigravity orthosis (*r* = 0.62, *p* = 0.01) and the neuroprosthetic condition (*r* = 0.58, *p* = 0.028).

**Table 2:** **Movement range of joints with and without stimulation**.

**Joint**	**Shoulder (°)**	**Upper arm (°)**	**Forearm (°)**	**Grip (pu)**
Movement in degrees (with and w/o stim)	18.2 ± 10.8 [1.7 54.4]	9.7 ± 4.9 [0.4 19.4]	8.9 ± 5.8 [0.2 29.2]	0.1 ± 0.1 [0.0 0.2]
Neuroprosthesis (with stimulation)	20.2 ± 11.9 [5.2 54.4]	11.0 ± 5.2 [2.7 19.4]	10.8 ± 9.2 [0.1 27.7]	0.1 ±0.1 [0.0 0.2]
Orthosis (w/o stimulation)	16.3 ± 9.7 [1.7 43.5]	8.4 ± 4.5 [0.4 16.1]	7.5 ± 4.9 [0.2 18.5]	0.1 ± 0.1 [0.0 0.2]
Significance level, *p*-value (^*^significant)	0.0012^*^	0.0002^*^	0.0007^*^	0.08 (not significant)

**Figure 6:**
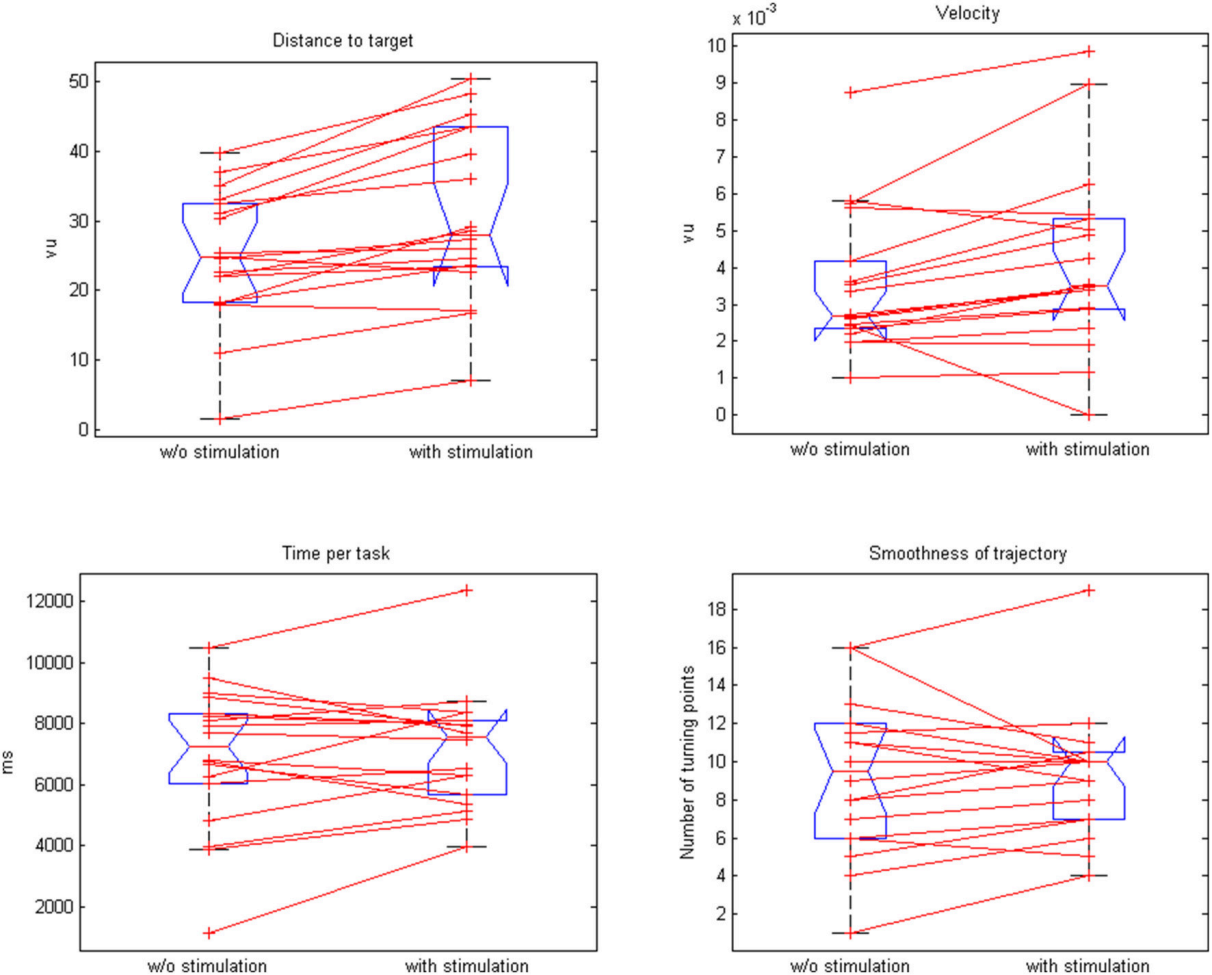
**Comparison of paired plots for mean values of distance to target, velocity, time per task, and smoothness of trajectories with and without stimulation**.

The neuroprosthesis increased the movement velocity (3.8 vs. 3.5 vu/s, *p* = 0.015) with a trend toward a faster task completion (6.9 vs. 7.2 s, *p* = 0.02) while preserving the smoothness of the trajectory (9.3 vs. 9.31, *p* = 0.46; Figure 6).

**Figure 7:**
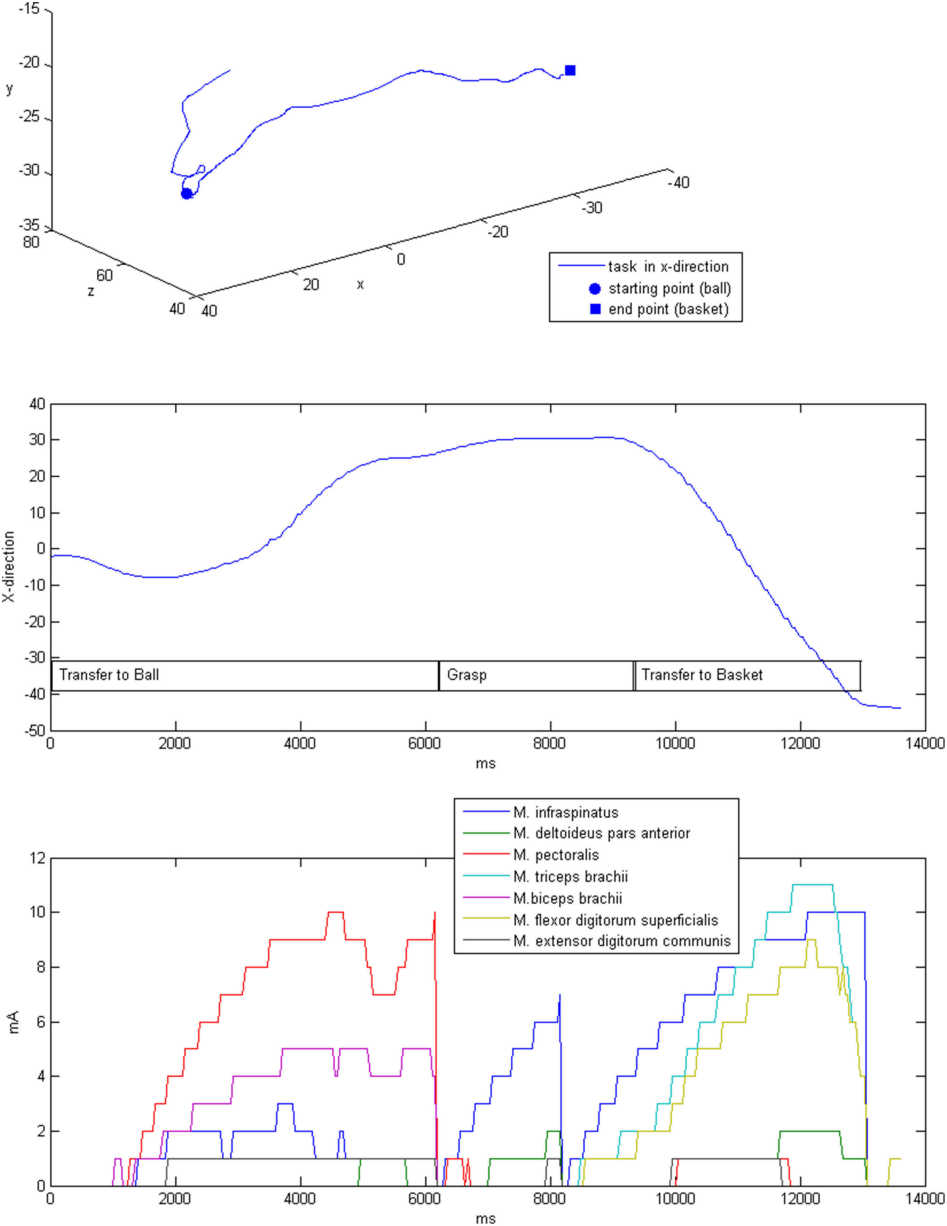
**Exemplary three-dimensional movement trajectory (upper row) with the respective movement in x-direction over time for different phases of the task (middle row)**. Stimulation intensities during the movement that were applied to the respective muscles (lower row).

The stimulation was applied after the self-induced movements of the patients by adapting the intensity in accordance with the output of the dynamic biomechanical arm model and the estimated target vector for each targeted muscle group (Table 3, Figure 7). Completion of the overall task took on average 6.9 s, while most of the time (mean 4.8 ± 0.6 s, i.e., 82% of the time) no or only minimal stimulation, i.e., <25% of Stim_max_, was applied. The same was true for the virtual transfer task of the ball into the basket, which was supported by minimal stimulation, i.e., <25% of Stim_max_, in 78% of the trajectory.The highest relative stimulation intensity was necessary to facilitate the grasping function, i.e., the grip strength in transfer movements, by applying stimulation intensities of >75% Stim_max_to the finger flexion muscle for about 22% of the time. The performance-dependent adaptation of stimulation resulted in a decrease in both stimulation intensity (on average by 35.4%) and period (on average by 36.77%) in the course of the session, which is suggestive of motor learning.

**Table 3:** **Stimulation parameters**.

**Muscle**	**M. flexor digitorum superficialis**	**M. extensor carpi radialis**	**M. biceps brachii**	**M. triceps brachii**	**M. pectoralis major**	**M. infraspinatus**	**M. deltoideus pars anterior**
Channel	1	2	3	4	5	6	7
Calibrated maximum stimulation intensity (mA)	9.67 [2.00 20.00]	8.8 [3 17]	11.4 [6 22]	11.7 [6 22]	8.9 [4 17]	9.8 [4 18]	8.7 [4 17]
Mean stimulation intensity (mA) and range	3.3 [0.8 6.3]	0.7 [0.3 1.3]	2.2 [0.9 4.7]	1.1 [0.4 2.0]	1.5 [0.6 4.1]	0.9 [0.4 2.1]	1.1 [0.4 2.5]
Stimulation period % (Amplitude [0% 25%])	48.6 [36.7 77.3]	88.4 [77.7 97.8]	69.9 [57.6 89.4]	87.6 [76.5 96.2]	71.2 [52.0 94.9]	84.2 [68.1 94.9]	79.4 [61.1 96.8]
Stimulation period % (Amplitude [25% 50%])	12.4 [0.0 21.8]	5.8 [1.2 14.5]	12.4 [6.0 18.5]	5.6 [2.4 8.5]	11.7 [3.6 22.4]	7.4 [2.9 16.8]	8.8 [1.6 19.0]
Stimulation period % (Amplitude [50% 75%])	16.9 [8.9 22.5]	3.5 [0.4 7.1]	9.9 [3.5 14.8]	4.0 [0.3 6.6]	10.0 [1.3 18.3]	4.5 [2.0 6.9]	5.7 [1.3 11.8]
Stimulation period % (Amplitude [75% 100%])	22.1 [5.6 40.3]	2.4 [0.4 5.2]	7.9 [0.6 17.5]	2.9 [0.2 8.5]	7.0 [0.2 13.0]	3.9 [0.2 8.9]	6.1 [0.3 13.3]

## Discussion

The present study demonstrated the feasibility of integrating multichannel closed-loop neuromuscular stimulation in an exoskeleton-based training; this neuroprosthesis increased the goal-oriented range of motion and movement velocity while preserving accuracy in chronic stroke patients with a severe impairment of the upper-extremity. The multi-joint exoskeleton for the paretic arm enabled the patients to perform task-oriented practice within a virtual environment (Housman et al., 2009), which they were unable to perform without assistance. Notably, unlike other studies with similarly affected stroke patients, in which robots completed a movement that the patients had begun (Klamroth-Marganska et al., 2014; Brauchle et al., 2015), this hybrid technology delivered antigravity-support only, i.e., provided no active assistance. Thus, the patient engagement was maximized by default in the present study, leaving no room for slacking; the continuous visual feedback of the arm kinematics enabled the patients to adjust their action online during each task; an approach closely resembling natural motor learning.

Such a closed-loop framework adheres to an operant conditioning rationale (Bauer et al., in press), providing contingent feedback to facilitate the targeted activity considered to be beneficial to recovery and which might ultimately lead to functional gain (Bauer and Gharabaghi, 2015a). One drawback of such restorative approaches, however, is that the considerable challenge of these exercises (Fels et al., 2015; Bauer and Gharabaghi, 2015b) might condition the patients to explore alternative, i.e., therapeutically undesirable strategies such as compensatory shoulder movements (Cirstea and Levin, 2000) or co-activation of non-targeted muscles (Gharabaghi et al., 2014b). Moreover, particularly in patients with severe impairments, non-successful trials may frustrate the participants, thereby compromising their motor learning.

In this context, adaptive neuromuscular stimulation, as applied in the present study, may support the exercises by extending the range of motion in accordance with the actual ability of each patient. More specifically, the range of motion correlated with the upper extremity Fugl-Meyer Assessment score of the patients for both the non-NMES and the NMES condition, indicating a targeted assistance of the genuine movement capability of each patient. Importantly, to avoid under-challenge, stimulation was applied adjunct to voluntary contraction and not as an alternative. Moreover, such an additive stimulation approach has proved effective in assisting reaching and grasping exercises in severely impaired, chronic stroke patients for repetitive task practice (Thrasher et al., 2008; Oujamaa et al., 2009; Mann et al., 2011). Unlike these previous approaches, however, our stimulation paradigm was (i) multi-channeled, i.e., targeting seven different muscles, (ii) model-based to follow the three-dimensional movement trajectory, (iii) performance-dependent to enhance task-oriented training, and (iv) subthreshold to avoid slacking:

- (i) Previous approaches combining functional electrical stimulation (FES) with mechanical support for the upper limb usually stimulated one or two muscles. Only recently, FES of thee joints, i.e., shoulder, elbow, and wrist, was implemented and shown to be effective in reducing upper limb impairment following stroke (Meadmore et al., 2014). Notably, more functional motor activities of the upper limb could be performed following this intervention: a finding that could not be achieved in an earlier study conducted by the same research group using exactly the same therapy dose (18 sessions, 60 min each) with FES to two proximal muscles only (Meadmore et al., 2012). Future studies will reveal whether a more fine-graded targeting of even more muscles—as shown to be feasible in the present study for stimulation-assisted task-oriented 3D exercises—leads to further functional gains when applied repetitively within a multi-session intervention.
- (ii) Conventional FES of the upper limb, even when physiologically triggered, follows an all-or-nothing concept. Only few research groups have explored model-based stimulation paradigms to precisely control FES for goal-oriented movements of the upper limb (Hughes et al., 2009; Meadmore et al., 2012, 2014). The most advanced approach used iterative learning control, which applied data from previous attempts in an effort to update the FES control signal on the current attempt (Meadmore et al., 2014). The reduction of error between real and reference trajectories within a biomechanical model thereby corresponded to improved performance over successive attempts. In addition, the supplied FES was reduced as performance improved to optimize motor learning (Meadmore et al., 2014). Our approach complements this strategy; instead of adjusting the stimulation from trial to trial, we tuned it within each trial. Rather than aiming to reduce the error between actual and reference trajectory with suprathreshold FES, we applied a ramping stimulation which, nevertheless, remained subthreshold throughout the task-oriented attempt (see below). Instead of one (Hughes et al., 2009), two (Meadmore et al., 2012), or three muscles (Meadmore et al., 2014), we integrated a total of seven muscles into our biomechanical model. By adding more muscles a larger number of movement directions could be addressed, thereby, covering a three-dimensional volume with movements in x-, y-, and z-direction. Despite these differences, the supplied multi-channel stimulation was reduced in our feasibility study as well. This could already be observed in the course of one session, suggesting that, even when applied subthreshold, an online adaptation of stimulation has immediate effects on motor learning.
- (iii) The performance-dependent stimulation applied in the present study was more subtle than in other approaches. Since no functional muscle contraction was achieved by the actual stimulation, *performance* was instead captured by gradual modulations of self-initiated, orthosis-assisted movements. It is therefore somewhat surprising that this novel approach resulted in task-adapted stimulation intensities for each of seven targeted muscles during the task-oriented exercises, e.g., with the highest relative stimulation intensity for facilitation of grip-strength occurring during transfer movements. This information about the different levels of assistance required by the muscles for specific goal-oriented tasks might be used in future studies to customize the exercises and training protocols in such a way as to target specific weaknesses, e.g., particular muscles/muscles groups in the course of a long-term training program.
- (iv) Neuromuscular stimulation alone, however, was not enough to facilitate reach-to grasp movements in our severely impaired patient group. Instead, multi-joint antigravity assistance was required to facilitate the task-oriented training in the 3D-virtual environment. Although, none of the targeted muscles was stimulated in a functionally relevant way to allow for overt muscle contraction, the cumulative effect of multi-channel subthreshold stimulation resulted in an increased range of motion and movement velocity while preserving smoothness during the goal-oriented exercises. This finding suggests a general facilitation of sensorimotor networks, which might provide a novel restorative approach in chronic stroke patients with a severe impairment of the upper-extremity. However, it has to be borne in mind that the applied ramping stimulation, based on a Boltzmann-fitting algorithm during each task, led to minimal stimulation intensity, i.e., <25% of Stim_max_, during most of the training period. Future studies need to explore whether different adaptive stimulation paradigms may achieve larger kinematic gains, e.g., by applying more neuromuscular stimulation, or by utilizing transcranial direct current stimulation to facilitate exoskeleton-based motor leaning (Naros et al., 2016b).

However, the current approach resulted in kinematic gains while still encouraging effort from the participants. To further generate a challenge for motor learning, the progression of training is necessary (Guadagnoli and Lee, 2004) and might be realized by reducing the FES support level (Meadmore et al., 2014) or by automated adaptation of training difficulty during robot-assisted stroke rehabilitation (Metzger et al., 2014). Both of these requirements are integrated into the presented neuroprosthetic set-up and need to be examined in more detail with regard to their respective clinical relevance in the targeted patient population by performing intervention studies with repetitive sessions.

The presented neuroprosthesis sparks hope for a general capacity for even larger gains, e.g., when additional interventions such as brain state-dependent cortical stimulation (Kraus et al., 2016a) are applied to maximally exploit the salvaged restorative potential. In particular, the task-related and muscle-specific facilitation provided by this hybrid device during reach-to-grasp exercises of severely impaired stroke patients, may deliver the framework for concurrent cortical stimulation. Activity-dependent transcranial magnetic stimulation, for example, may constitute such an additional input during robot-assisted training (Gharabaghi, 2015; Massie et al., 2015). Associative brain state-dependent stimulation (Royter and Gharabaghi, 2016) during brain-robot interface exercises has the potential to unmask latent corticospinal connectivity after stroke (Gharabaghi et al., 2014a). The application of such state-dependent stimulation synchronized to maximum gains of assisted range of motion may consolidate the involved corticospinal circuits in accordance with Hebbian-like plasticity rules. More specifically, neuroprosthetic exercises based on brain-robot feedback may result in connectivity changes of cortico-cortical motor networks (Vukeliæ et al., 2014; Vukelić and Gharabaghi, 2015a,b) and lead to a re-distribution of cortico-spinal connections (Kraus et al., 2016b). This advanced assistive rehabilitation technology may thereby constitute a back-door to the motor system to further improve the scope for recovery (Bauer et al., 2015).

In summary, combining robotic assistance with adaptive closed-loop neuromuscular stimulation provides customized rehabilitation environments for severely impaired stroke patients, and may increase kinematic parameters while preserving the voluntary effort of patients, during rehabilitation training. Whether these technological refinements also lead to relevant functional gains requires investigation in controlled intervention studies in comparison to dose-matched, conventional physiotherapy.

## Author contributions

FG participated in the study design, software development, data acquisition and analysis. AG participated in the study design and data analysis. Authors jointly drafted and approved the final manuscript.

### Conflict of interest statement

The authors declare that the research was conducted in the absence of any commercial or financial relationships that could be construed as a potential conflict of interest.
